# ETV2-TET1/TET2 Complexes Induce Endothelial Cell-Specific Robo4 Expression via Promoter Demethylation

**DOI:** 10.1038/s41598-018-23937-8

**Published:** 2018-04-04

**Authors:** Toru Tanaka, Kohei Izawa, Yusuke Maniwa, Maki Okamura, Atsumasa Okada, Tomoko Yamaguchi, Keisuke Shirakura, Naoki Maekawa, Hayato Matsui, Kenji Ishimoto, Nobumasa Hino, Osamu Nakagawa, William C. Aird, Hiroyuki Mizuguchi, Kenji Kawabata, Takefumi Doi, Yoshiaki Okada

**Affiliations:** 1 Graduate School of Pharmaceutical Sciences, Osaka University, Suita City, Osaka, 565-0871 Japan; 2 Laboratory of Stem Cell Regulation, National Institutes of Biomedical Innovation, Health and Nutrition, Ibaraki City, Osaka, 567-0085 Japan; 3 Department of Molecular Physiology, National Cerebral and Cardiovascular Center Research Institute, Suita City, Osaka, 565-8565 Japan; 4 Center for Vascular Biology Research and Division of Molecular and Vascular Medicine, Beth Israel Deaconess Medical Center, Boston, MA 02215 USA

## Abstract

Although transcription factors regulating endothelial cell (EC)-specific gene expression have been identified, it is not known how those factors induce EC-specificity. We previously reported that DNA hypomethylation of the proximal promoter elicits EC-specific expression of Roundabout4 (Robo4). However, the mechanisms establishing EC-specific hypomethylation of the Robo4 promoter remain unknown. In this study, we demonstrated that the hypermethylated Robo4 proximal promoter is demethylated as human iPS cells differentiate into endothelial cells. Reporter assays demonstrated that ETV2, an ETS family transcription factor, bound to ETS motifs in the proximal promoter and activated Robo4 expression. Immunoprecipitation demonstrated direct interaction between ETV2 and methylcytosine-converting enzymes TET1 and TET2. Adenoviral expression of ETV2-TET1/TET2 complexes demethylated the Robo4 promoter and induced Robo4 expression in non-ECs. In summary, we propose a novel regulatory model of EC-specific gene expression via promoter demethylation induced by ETV2-TET1/TET2 complexes during endothelial differentiation.

## Introduction

To determine the underling mechanisms of tissue-specific gene expression, various genes have been studied. Endothelial cell (EC)-specific genes have been shown to be regulated by transcription factors, including specificity protein 1 (SP1), ETS proteins such as ETV2, FLI1, ERG, and ETS1^1,2^, Group F Sry-related high-mobility box factors (SOX7, −17, and −18), and vascular endothelial zinc finger 1^3^. Among these, ETV2 is essential for development of EC and hematopoietic cells^4,5^ and directly reprograms fibroblasts into ECs^6,7^. SOX F and VEZF1 in progenitor cells regulate EC function during embryogenesis^8,9^. Although these factors have been shown to play essential roles during EC differentiation, it remains unclear whether these regulate EC-specific gene expression.

Recent reports demonstrate that tissue-specific gene expression is regulated via epigenetic mechanisms, including DNA methylation^10^. In vertebrates, methylation is catalyzed by DNA methyltransferase, which transfers a methyl group to the C-5 atom of cytosine in a CpG dinucleotide to facilitate gene suppression in cellular processes such as X chromosome inactivation^11^. Conversely, DNA demethylation induces transcription^12,13^, and is regulated by ten-eleven translocation 1–3 (TET1-3), which oxidizes 5-methylcytosine to 5-hydroxymethylcytosine, 5-formylcytosine, and 5-carboxylcytosine. These intermediates are then converted to unmodified cytosine by active or passive demethylation mechanisms^14,15^. Additionally, transcription factors such as PPARγ, NANOG, PRDM14, and PU.1 were recently demonstrated to directly or indirectly interact with TET1 and/or TET2 to elicit demethylation^16–19^. In line with this model, the proximal promoters of several EC-specific genes are hypomethylated in ECs, but hypermethylated in non-ECs^20^. However, the mechanisms by which these promoters are specifically hypomethylated in ECs has not been established.

To investigate the mechanisms of EC-specific gene expression, we have been studying an EC-specific gene, Roundabout4 (Robo4)^21^. Robo4 is a transmembrane protein that stabilizes vasculature in pathological angiogenesis by suppressing EC migration, proliferation, and hyperpermeability induced by vascular endothelial growth factor (VEGF)^22–24^. Recently, Robo4 has been shown to regulate cytokine production in inflammation^25^. Robo4 expression is driven by a 3 kb promoter activated by transcription factors such as GA-binding protein (GABP), SP1, AP-1, NF-κB, SOX7, and SOX18^26–30^. The Robo4 proximal promoter is hypomethylated in ECs and hypermethylated in non-ECs^31^. This hypermethylation suppresses Robo4 expression by inhibiting SP1 binding to the proximal promoter, and thereby helps restrict expression to ECs, indicating that EC-specific Robo4 expression is regulated by DNA methylation. However, it remains unclear how the Robo4 proximal promoter is specifically demethylated in ECs.

In this study, we investigated how methylation of the endogenous Robo4 promoter in human induced pluripotent stem (iPS) cells is altered during differentiation into ECs. We demonstrate that the highly methylated Robo4 promoter is demethylated during cell differentiation and that this demethylation is regulated by ETV2-TET1/TET2 complexes. Based on these data, we propose a novel regulatory mechanism of EC-specific gene expression.

## Results

### Robo4 Promoter Is Demethylated During Differentiation of iPS Cells into ECs

**Figure 1:**
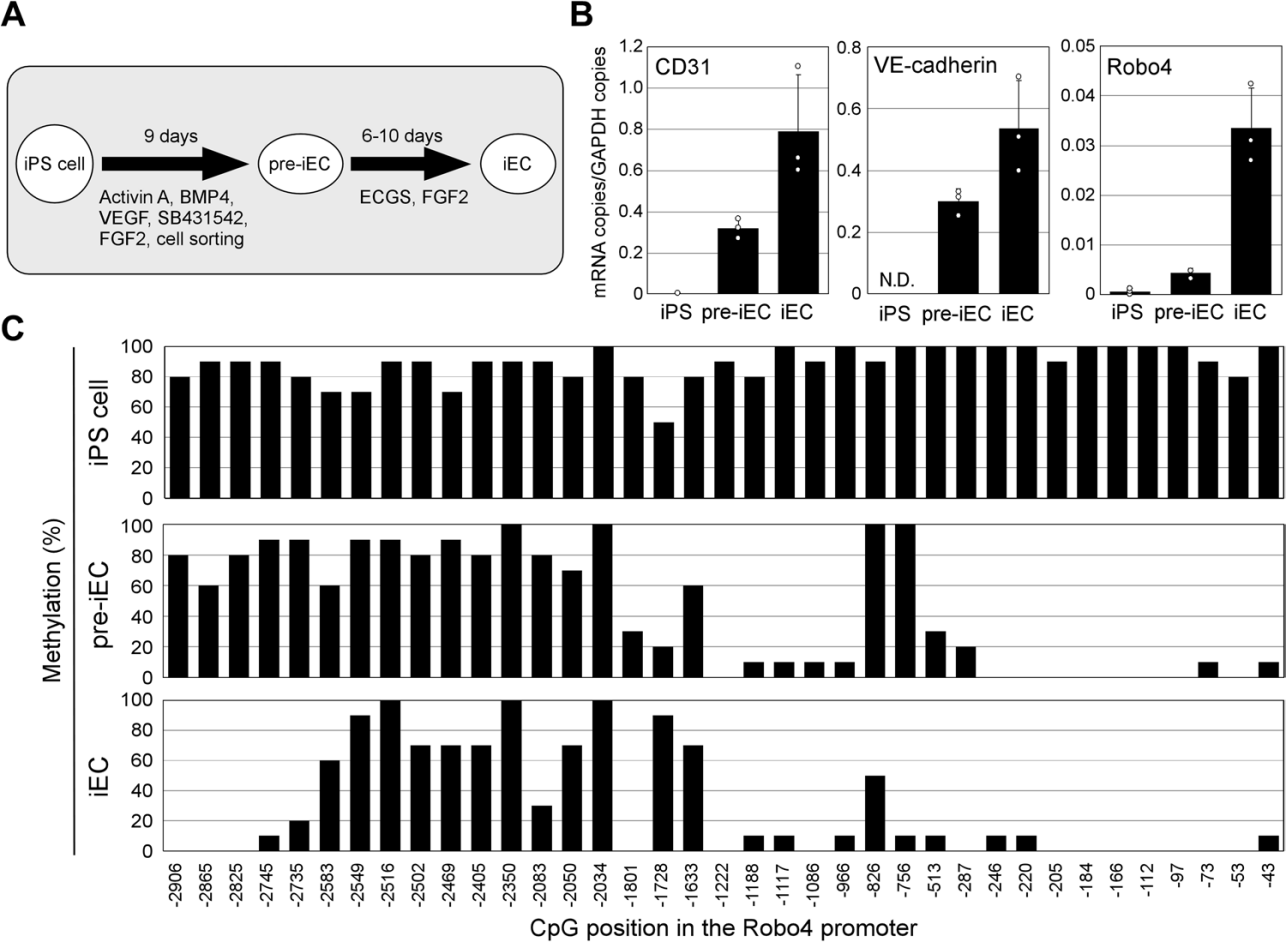
Demethylation of the Robo4 promoter during differentiation of iPS cells into ECs. (**A**) Differentiation of human iPS cells into ECs. iPS cells were differentiated into pre-mature (pre-iECs) and mature ECs (iECs). (**B**) Expression of EC-specific genes in iPS-derived cells. Expression of CD31, VE-cadherin and Robo4 mRNA in iPS cells, pre-iECs, and iECs were measured by real-time RT-PCR. Data are means ± S.D. (n = 3). (**C**) Methylation patterns of the Robo4 promoter in iPS-derived cells. CpG methylation of the Robo4 promoter in iPS cells, pre-iECs, and iECs was analyzed by bisulfite sequencing. Each graph indicates the CpG position in the promoter and the percentage of methylated CpG.

To investigate methylation of the human Robo4 promoter, human iPS cells were differentiated into pre-mature (pre-iECs) and mature ECs (iEC) (Fig. 1A). Real-time RT-PCR of transcripts from these cells showed a gradual increase of EC markers, including CD31, VE-cadherin and Robo4, as iPS cells differentiated into ECs (Fig. 1B). We then isolated genomic DNA from these cells, and analyzed methylation of the Robo4 promoter by bisulfite sequencing (Fig. 1C, Supplementary Fig. S1). In iPS cells, the promoter was highly methylated throughout. However, regions within −1.5 kb of the transcription start site were almost completely demethylated in pre-iECs, with the exception of sites at −826 and −756. Further demethylation of sequences between −2906 and −2735 was observed in iECs. Collectively, these data demonstrated that the Robo4 promoter is demethylated at specific positions during differentiation.

### ETV2 Potentially Demethylates the Robo4 Proximal Promoter

**Figure 2:**
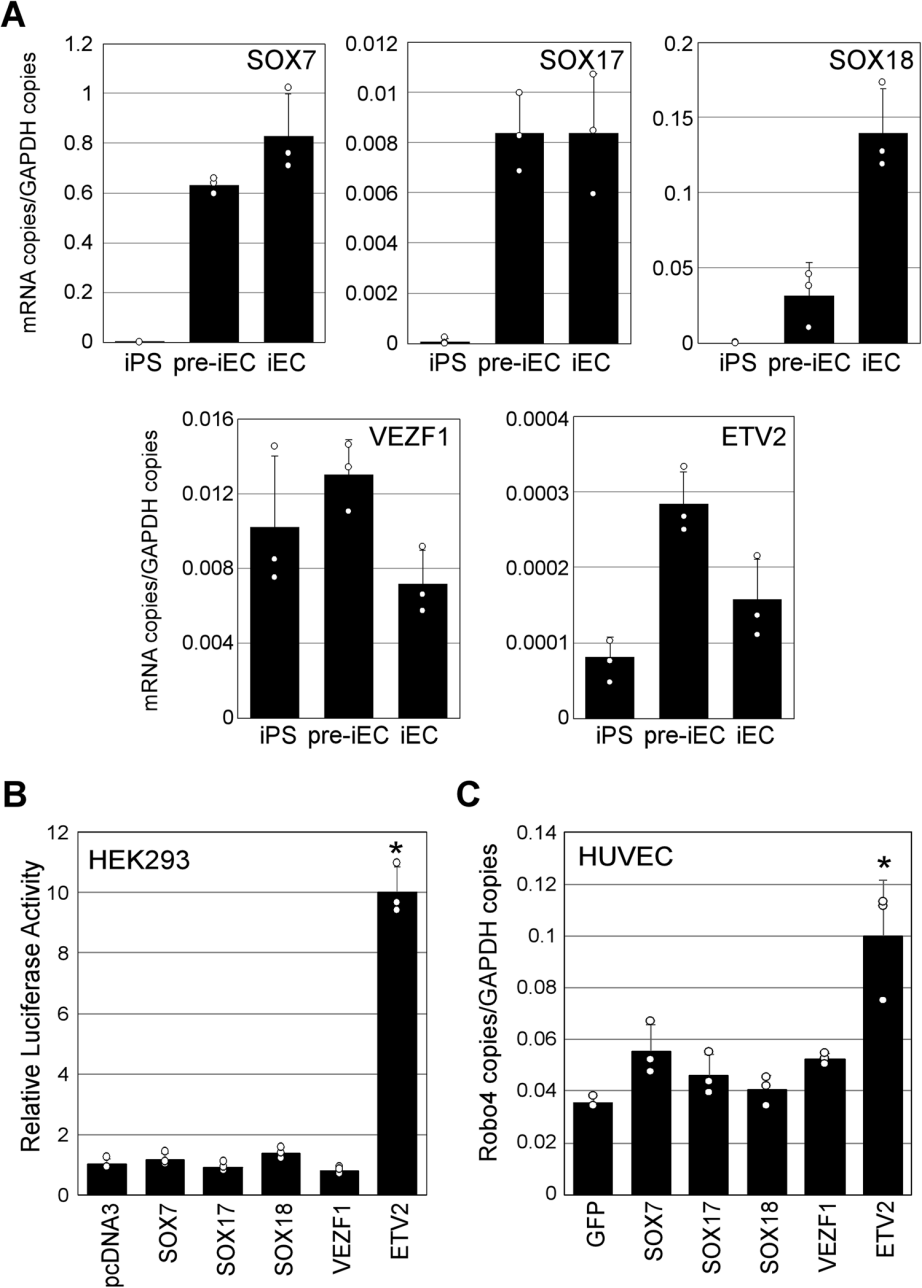
Effect of transcription factors on Robo4 promoter activity in iPS-derived cells. (**A**) Expression of transcription factors in iPS-derived cells. Expression of SOX factors, VEZF1, and ETV2 in iPS-derived cells was measured by real-time RT-PCR. Data are means ± S.D. (n = 3). (**B**) Effects of transcription factors on Robo4 promoter activity. HEK293 cells were co-transfected with a Robo4 promoter-luciferase reporter construct and expression vectors for transcription factors or GFP, and luciferase activity was measured 24 h after transfection. Data are means ± S.D. (n = 3). *p < 0.05 by two-tailed Dunnett’s test vs pcDNA3. (**C**) Effect of transcription factors on Robo4 expression in ECs. Transcription factors or GFP were expressed from adenovirus vectors in HUVECs, and Robo4 expression was measured after 48 h by real-time RT-PCR (n = 3). *p < 0.05 by two-tailed Dunnett’s test vs GFP.

Since the Robo4 promoter is demethylated as iPS cells differentiate into pre-iECs, we investigated by real-time RT-PCR the expression of transcription factors during differentiation, including SOX7, SOX17, SOX18, VEZF1, and ETV2 (Fig. 2A). Expression of SOX7, SOX17, and SOX18 strongly increased during differentiation, while expression of VEZF1 and ETV2 peaked in pre-iECs and diminished in iECs. Hence, expression of all five factors coincides with, and perhaps mediates, Robo4 promoter demethylation.

Each transcription factor was then co-transfected into HEK293 cells with a reporter construct driven by the Robo4 promoter. Expression of ETV2, but not other factors, significantly increased promoter activity (Fig. 2B). Accordingly, adenoviral overexpression of ETV2, but not other factors, significantly increased Robo4 expression in human umbilical vein endothelial cells (HUVECs) (Fig. 2C, Supplementary Fig. S2). These results indicated that ETV2 regulates Robo4 expression, and presumably drives promoter demethylation.

### ETV2 Activates Robo4 Promoter via Four ETS motifs in the Proximal Region

**Figure 3:**
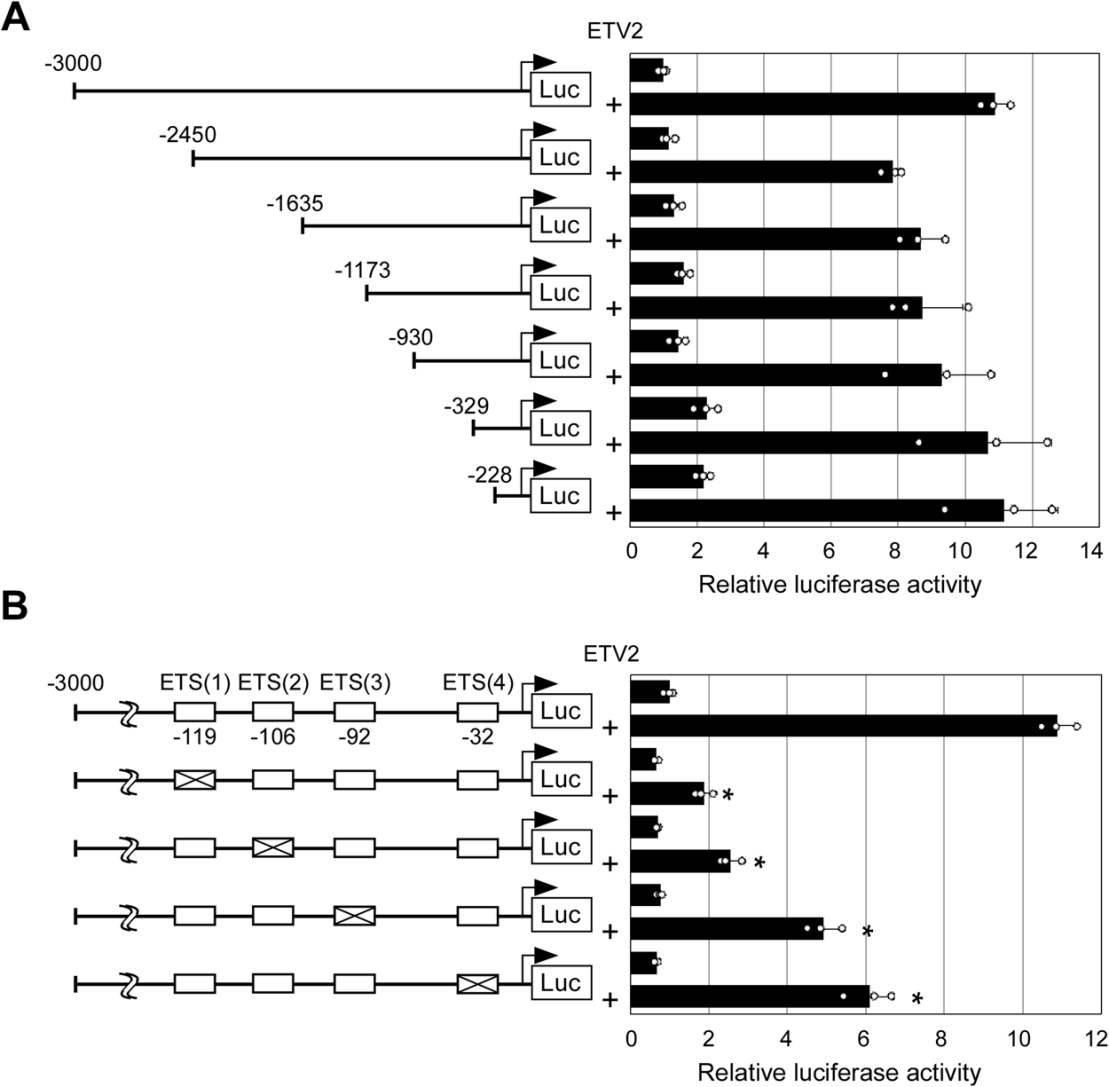
ETV2 response elements in the Robo4 promoter. (**A**) Effect of ETV2 on truncated Robo4 promoter activity. Wild type and truncated Robo4 promoters were analyzed with or without ETV2 by luciferase reporter assays in HEK293 cells. Numbers indicate distance (base) from the transcription start site. Data are means ± S.D. (n = 3). (**B**) Effect of ETS motifs on ETV2-mediated Robo4 promoter activation. Wild type and Robo4 promoters with point mutations in ETS motifs were analyzed with or without ETV2 by luciferase reporter assays in HEK293 cells. Data are means ± S.D. (n = 3). *p < 0.05 by two-tailed Dunnett’s test vs wild type.

To identify Robo4 promoter elements that respond to ETV2 interaction, reporter assays were performed in HEK293 cells co-transfected with ETV2 and reporter constructs driven by truncated promoters (Fig. 3A). Both full-length and truncated promoters were activated by ETV2 to comparable levels, suggesting that ETV2-responsive elements are within −228 bp of the transcription start site. Since we have previously identified the evolutionarily-conserved ETS-binding motifs ETS(1), ETS(2), ETS(3), and ETS(4) at −119, −106, −92, and −32, respectively, we investigated whether these motifs are required for ETV2-dependent promoter activation (Fig. 3B). Mutations in these motifs decreased promoter activity to 17%, 23%, 45%, and 56% of wild type, respectively, demonstrating that ETV2 activated the Robo4 promoter via all four motifs. Notably, the importance of each motif was correlated with similarity to the ETV2 consensus motif (Supplementary Fig. S3).

### ETV2 Binds to the Four ETS motifs in the Proximal Promoter

**Figure 4:**
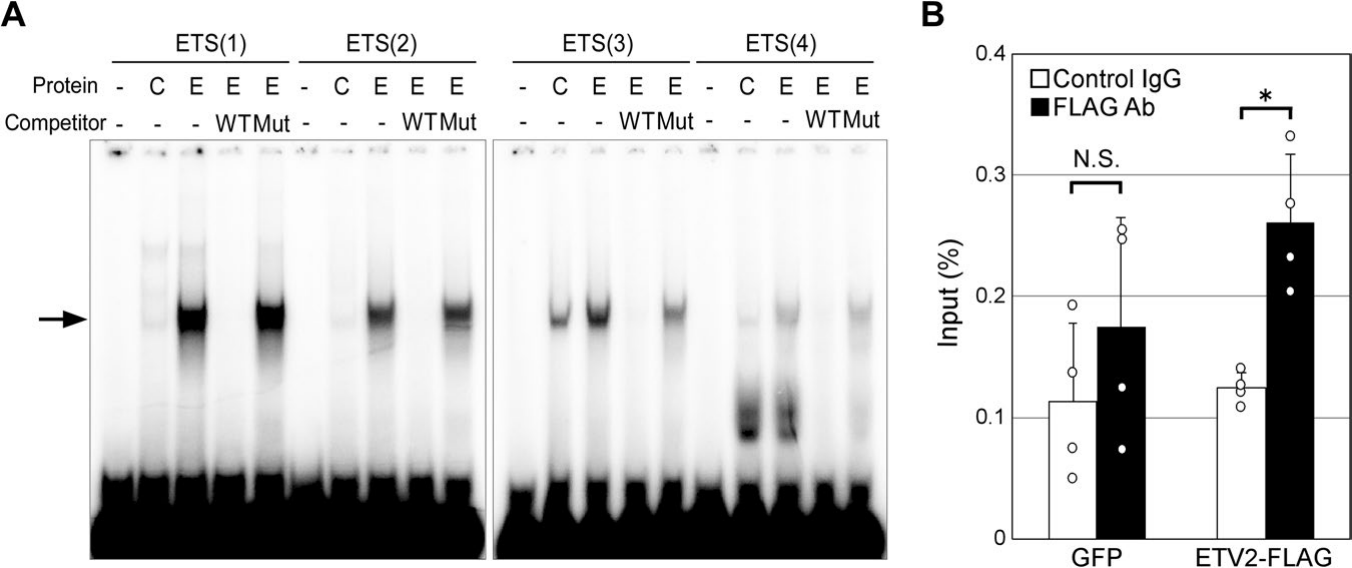
ETV2 binding to ETS motifs in the Robo4 promoter. (**A**) ETV2 binding to ETS motifs in Robo4 promoter. Electrophoretic mobility shift assay using ETV2 (E), control protein (C) and ^32^P-labeled probes containing ETS motifs. A 50-fold molar excess of unlabeled wild type (WT) or mutant (Mut) ETS motif was used as competitor. The arrow indicates the shifted bands derived from an ETV2-DNA complex. (**B**) ETV2 binding to endogenous Robo4 promoter. Chromatin immunoprecipitation with anti-FLAG or control IgG of HUVECs expressing ETV2-FLAG or GFP. Immunoprecipitated DNA fragments were analyzed by real-time PCR targeting the Robo4 promoter. Data are means ± S.D. (n = 4). *p < 0.05 by two-tailed Student’s t-test vs control IgG.

Electrophoretic mobility shift assays were performed to investigate whether ETV2 binds to the four ETS motifs in the Robo4 promoter. ETV2, but not control protein, shifted the mobility of all four radiolabeled probes (Fig. 4A). The shift was most pronounced with ETS(1), followed by ETS(2), ETS(3), and ETS(4) (in that order). All interactions were abolished by corresponding wild type competing probes, but not by mutated competing probes.

To confirm the interaction between ETV2 and Robo4 promoter elements, HUVECs expressing ETV2-FLAG or GFP from an adenovirus vector were analyzed by chromatin immunoprecipitation (ChIP). In comparison to control IgG, significantly more Robo4 promoter was precipitated with FLAG antibody from cells expressing ETV2-FLAG, but not from cells expressing GFP (Fig. 4B). Taken together, these results indicated that ETV2 binds to the endogenous Robo4 promoter via ETS motifs.

### ETV2 Interacts with TET1 and TET2 that are expressed during EC Differentiation

**Figure 5:**
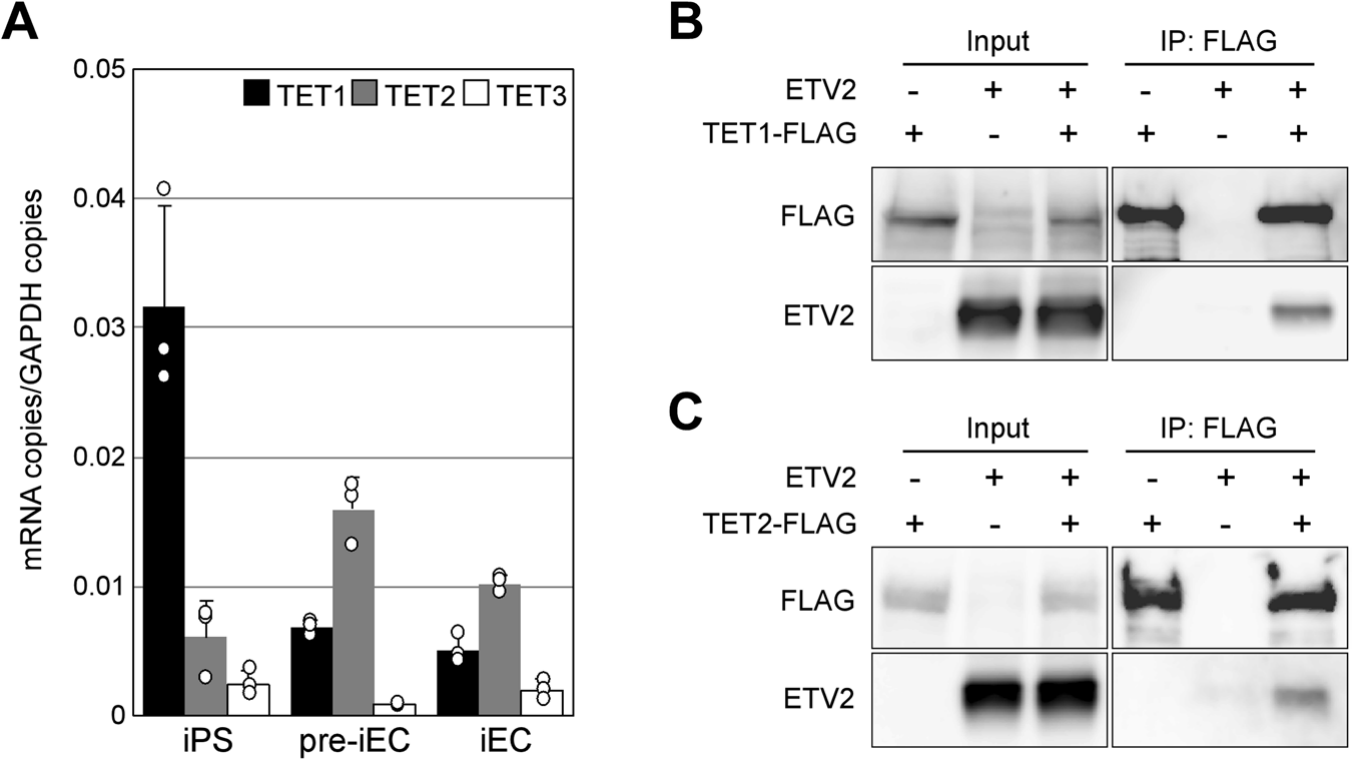
Expression of TET proteins in iPS-derived cells, and their interaction with ETV2. (**A**) Expression of TET1-3 in iPS-derived cells. Expression of TET1-3 mRNAs in iPS cells, pre-iECs, and iECs were measured by real-time RT-PCR. Data are means ± S.D. (n = 3). (**B**,**C**) Interactions between ETV2 and TET1/TET2. Co-immunoprecipitation with FLAG antibody of COS-7 cells infected with adenovirus vectors for ETV2 and FLAG-tagged TET1/TET2. The input and eluate were analyzed by western blot with antibodies against FLAG and ETV2.

We then tested whether ETV2 directly interacts with TET demethylases. We first investigated by real-time RT-PCR the expression of TET1-3 during differentiation of iPS cells (Fig. 5A). TET1 was abundantly expressed in iPS cells, but diminished with differentiation into iECs. Conversely, TET2 was expressed at low levels in iPS cells, but spiked in pre-iECs and slightly decreased in iECs. In contrast, TET3 was barely expressed. These results indicated that TET1 and TET2 (TET1/TET2) are adequately expressed as iPS cells differentiate into pre-iECs. To investigate interaction between ETV2 and TET1/TET2, COS-7 cells were infected with adenovirus vectors containing TET1-FLAG and ETV2, and immunoprecipitated with anti-FLAG (Fig. 5B). Western blotting indicated that TET1-FLAG co-precipitated with ETV2. Similar results were obtained from COS-7 cells expressing TET2-FLAG and ETV2 (Fig. 5C). Collectively, the data indicated that ETV2 interacts with both TET1 and TET2.

### ETV2 and TET1/TET2 complexes synergistically demethylate the proximal promoter

**Figure 6:**
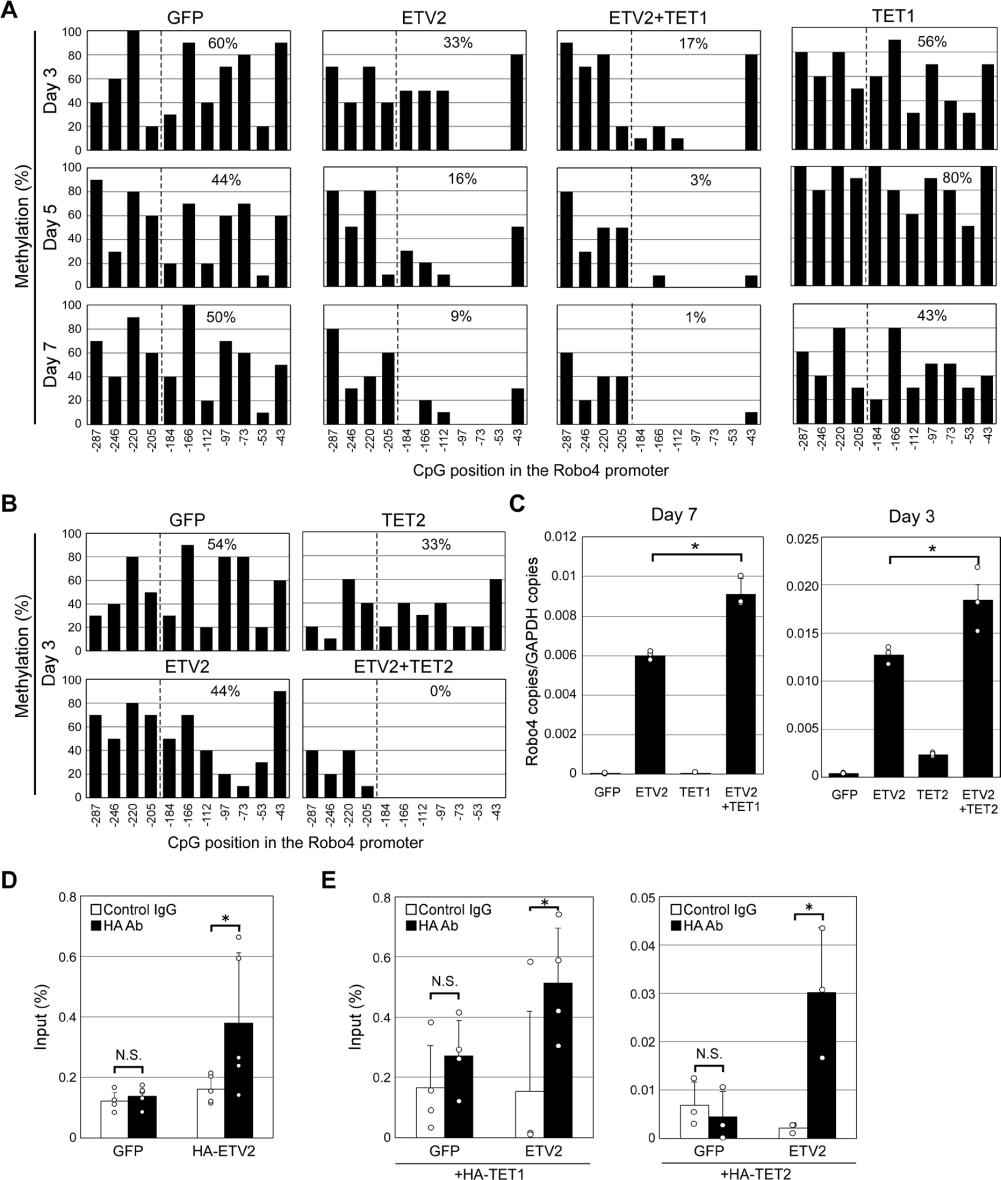
Demethylation of the Robo4 promoter by ETV2 and TET1/TET2. (**A**,**B**) Methylation patterns of Robo4 proximal promoter in fibroblasts expressing ETV2 and/or TET1/TET2. Fibroblasts were infected with adenovirus vectors at days 0 and 3 to express either GFP or ETV2 with or without TET1/TET2, and cultured for 3–7 days. Methylation of the Robo4 promoter was analyzed by bisulfite sequencing. Data are % methylated CpGs within 200 bp upstream of the transcription start site. (**C**) Effect of ETV2 and TET1/TET2 on Robo4 expression in fibroblasts. Fibroblasts were infected with adenovirus vectors to express GFP or ETV2 with or without TET1/TET2, and cultured for 3–7 days.. Expression of Robo4 was measured by real-time RT-PCR. Data are means ± S.D. (n = 3). *p < 0.05 by two-tailed Tukey-Kramer test. (**D**) Binding of ETV2 to endogenous Robo4 proximal promoter in fibroblasts. Chromatin immunoprecipitation was performed using anti-HA antibody and human dermal fibroblasts expressing HA-ETV2. Immunoprecipitated DNA was analyzed by real-time PCR targeting the Robo4 proximal promoter. Data are means ± S.D. (n = 5). *p < 0.05 by two-tailed Student’s t-test vs control IgG. (**E**) ETV2-dependent TET1/2 binding to the endogenous proximal promoter. Chromatin immunoprecipitation was performed using fibroblasts expressing HA-TET1 or HA-TET2 with or without ETV2. Data are means ± S.D. (n = 4 for HA-TET1, n = 3 for HA-TET2). *p < 0.05 by two-tailed Student’s t-test vs control IgG.

To investigate whether ETV2 and TET1/TET2 demethylate the Robo4 promoter, we overexpressed ETV2 or GFP (as control) with or without TET1 in human dermal fibroblasts, in which the endogenous Robo4 promoter is methylated^31^. The Robo4 promoter was highly methylated on day 3 in control cells (Fig. 6A, Supplementary Fig. S4A). However, expression of ETV2 decreased methylation, and co-expression of TET1 enhanced this effect. Expression of TET1 alone did not affect methylation. Synergistic demethylation by ETV2 and TET1 was even more pronounced on days 5 and 7. Similarly, ETV2 and TET2 also cooperatively demethylated the Robo4 promoter even on day 3 (Fig. 6B, Supplementary Fig. S4B). Collectively, the data indicated that ETV2 and TET1/TET2 synergistically demethylate the Robo4 promoter. Robo4 expression was then measured by real-time PCR in fibroblast cells expressing GFP or ETV2 with or without TET1/TET2 (Fig. 6C, Supplementary Fig. S5). Expression of ETV2 by itself significantly induced Robo4 expression, while expression of TET1 or TET2 alone did not. Co-expression of ETV2 and TET1/TET2 further increased Robo4 expression. Taken together, the data demonstrated that ETV2 and TET1/TET2 induce Robo4 expression via promoter demethylation, and suggested that along with ETV2 co-expression, TET2 demethylated the Robo4 promoter more efficiently than TET1 did.

To investigate if promoter demethylation is induced by ETV2 and ETV2-TET1/TET2 complexes bound to the Robo4 promoter, ChIP was performed in fibroblasts expressing ETV2 and TET1/TET2 with or without HA-tag. In cells expressing HA-ETV2, HA-ETV2 was bound to the Robo4 promoter (Fig. 6D). In cells expressing HA-TET1 or HA-TET2 with or without ETV2 co-expression, both TET1 and TET2 were bound to the promoter only when ETV2 was co-expressed (Fig. 6E). These results indicated that ETV2 and ETV2-TET1/TET2 complexes bound to the methylated promoter in fibroblasts.

### ETV2 but no other endothelial ETS proteins interact with TET1

**Figure 7:**
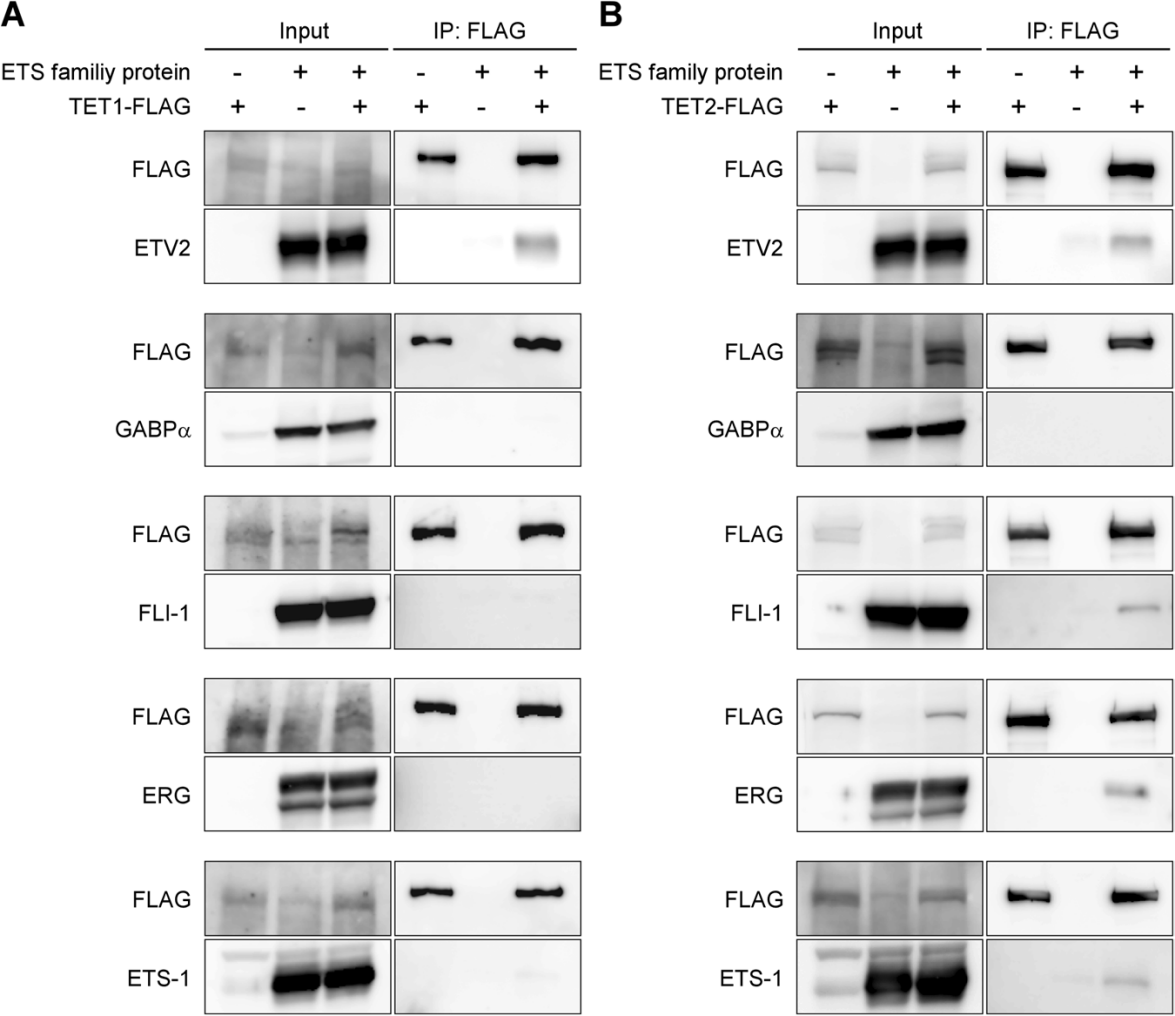
Interaction between TET1/TET2 and endothelial ETS proteins. (**A**,**B**) Co-immunoprecipitation with FLAG antibody and COS-7 cells transfected with expression vectors for each ETS proteins and infected with adenovirus vectors for TET1-FLAG (**A**) and TET2-FLAG (**B**). Proteins in the input and eluate were analyzed using western blotting with antibodies against FLAG and ETS proteins, including ETV2, GABPα, FLI-1, ERG, and ETS-1.

To investigate whether other endothelial ETS proteins bind to TET1/TET2, immunoprecipitation assays were performed with COS7 cells expressing GABPα, FLI-1, or ERG, and ETS-1 (Fig. 7). TET1 interacted with none of these factors, while TET2 interacted with FLI-1, ERG, and ETS-1, but not GABPα, which has been shown to bind to the Robo4 proximal promoter. These observations indicated different binding properties of ETS proteins to the TET1 and TET2, and suggested the unique function of ETV2 among ETS proteins.

## Discussion

In the current study, we investigated the mechanisms underlying regulation of EC-specific Robo4 expression. We now demonstrate for the first time that ETV2 and TET1/TET2 complexes bind to and demethylate the Robo4 promoter, and induce EC-specific Robo4 expression during differentiation. Consistent with previous reports^32^, ETV2 expression is observed temporarily in pre-iEC cells during differentiation. ETV2 binds to the ETS motifs in the proximal promoter, including the ETS(1) motif that binds to GABPα in primary ECs^26,27^. Furthermore, GABPα did not interact with TET1/TET2. Therefore, we propose a novel mechanism of EC-specific Robo4 gene expression (Supplementary Fig. S6). In this model, ETV2 is expressed as iPS cells differentiate into pre-iECs, interacts with TET1/TET2, binds to ETS motifs, and demethylates the Robo4 promoter to induce expression. As cells further differentiate into ECs, ETV2 expression diminishes, and GABPα is recruited to the ETS(1) motif to stimulate and sustain Robo4 expression. This is the first evidence regarding gene regulation by ETV2 and GABPα thorough the same ETS motif.

Accumulating evidence indicates that ETV2 is a master regulator of EC-specific gene expression and EC differentiation. However, among several ETS family proteins expressed in ECs, the reason for ETV2 alone to function as a master regulator is not yet understood. To further elucidate this, we analyzed whether TET1/TET2 bind to other endothelial ETS proteins, including GABPα, FLI-1, ERG, and ETS-1. Surprisingly, TET1 interacted with none of these factors, whereas TET2 interacts with FLI-1, ERG, and ETS-1, but not with GABPα. This suggested that ETV2 functions as a master regulator because of its unique ability to interact with TET1. In other words, ETV2 interacts with TET1 and demethylates the promoter at an early stage of EC differentiation when TET1 is highly expressed (Fig. 5A). In contrast, FLI-1, ERG, and ETS-1 interact with TET2 and may regulate demethylation and expression of other EC-specific genes at later stages when TET2 is predominantly expressed. Thus, these data suggested that multiple ETS proteins regulate EC-specific gene expression through interactions with TET1/TET2.

We observed differences in demethylation activity between TET1 and TET2. Along with ETV2 co-expression, TET2 demethylated the Robo4 proximal promoter more rapidly than TET1 did. In addition, our ChIP assay detected relatively stronger signal indicating binding of the ETV2-TET2 complex to the promoter compared to the signal for the ETV2-TET1 complex. Therefore, we speculated that, as compared to the ETV2-TET1 complex, the ETV2-TET2 complex stably binds to the Robo4 promoter and strongly demethylates the promoter. Further study on ETV2-TET1/TET2 complexes is needed to better understand the functional differences between TET1 and TET2.

ETV2 was recently shown to directly reprogram fibroblasts into ECs^7^. Our data and those of others suggest a potential mechanism of this reprogramming, in which exogenous expression of ETV2 promotes demethylation of the Robo4 promoter and induces Robo4 expression in fibroblasts. This process is probably mediated by endogenous TET2, although exogenous expression of TET1/TET2 enhanced promoter demethylation and Robo4 expression. Recent reports suggest that other EC-specific genes are also regulated by similar mechanisms. For example, the proximal promoters of PECAM-1, eNOS, von Willebrand factor, and VE-cadherin are also specifically hypomethylated in ECs but methylated in non-ECs^20^. Genome-wide surveys identified ETV2-binding sites in the proximal promoters of FLK1, VE-cadherin, and TIE2^6,32^. In addition, ETV2 and TET1/TET2 synergistically increased expression of VE-cadherin, as well as Robo4 in fibroblasts (Supplementary Fig. S5). These data suggested that ETV2 and TET1/TET2 complexes might regulate promoter demethylation, induce expression of other EC-specific genes, and finally convert fibroblasts into ECs. Genome wide analyses of the promoters of other ETV2 target genes may suggest the direct mechanisms underlying this regulation.

## Methods

### Human iPS Cell Culture and Differentiation

Human iPS cells (201B7, provided by Dr. S. Yamanaka) were maintained on mitomycin C-treated mouse embryonic fibroblasts in ReproStem medium (ReproCELL, Yokohama, Japan) supplemented with 5 ng/mL fibroblast growth factor 2 (FGF2). The cells were then differentiated into functional ECs according to a previous report^33^, with some modification. Briefly, iPS cells were dissociated with Accutase (Merck-Millipore, Billerica, MA), and resuspended in differentiation medium, which consists of StemPro-34 medium (Life Technologies, Carlsbad, CA) enriched with StemPro-34 Nutrient Supplement (Life Technologies), 0.45 mmol/L 1-thioglycerol, 50 μg/mL ascorbic acid, 2 mmol/L L-glutamine, 120 μg/mL streptomycin, and 200 μg/mL penicillin) supplemented with 20 ng/mL bone morphogenetic protein 4 (BMP4), 2 ng/mL Activin A, and 10 μmol/L Y27632. Cells were then seeded on a Lipidure-coated plate (Thermo Scientific, Waltham, MA), and cultured for two days. The resulting embryoid bodies were cultured for another two days in differentiation medium supplemented with 20 ng/mL BMP4 and 5 ng/mL vascular endothelial growth factor (VEGF). Subsequently, embryoid bodies were resuspended in differentiation medium supplemented with 20 ng/mL BMP4, 5 ng/mL VEGF, and 10 μmol/L SB431542. The resulting cells were cultured on a 6 cm petri dish for two days, and the medium was then replaced with differentiation medium supplemented with 20 ng/mL VEGF and 5 ng/mL FGF2. CD34^+^CD144^+^ cells (pre-iECs) in the resulting cells were purified using an SH-800 cell sorter (Sony, Tokyo, Japan), and cultured on fibronectin-coated plates in differentiation medium supplemented with 100 mg/mL heparin, 20 ng/mL FGF2, and 100 mg/mL EC growth supplement (Sigma, St. Louis, MO). To prepare mature ECs (iECs), pre-iECs were cultured for 6–10 days until confluency.

### Cell Culture

HUVECs and human dermal fibroblasts were purchased from Lonza (Basel, Switzerland). HUVECs were cultured in EGM-2-MV media (Lonza), while human dermal fibroblasts were cultured in FGM-2 media (Lonza). Human embryonic kidney cells (HEK293 cells) and African Green monkey SV40-transfected kidney fibroblast cells (COS-7 cells) were cultured in Dulbecco’s modified Eagle’s medium supplemented with 10% fetal bovine serum, 100 IU/mL penicillin, and 100 µg/mL streptomycin. All cells were cultured at 37 °C and 5% CO_2_.

### Bisulfite Sequencing

Genomic DNA was extracted using ISOGEN reagent (Nippon Gene, Tokyo, Japan) from undifferentiated and differentiated iPS cells, and from human dermal fibroblasts infected with 10,000 adenovirus particles/cell on days 0 and 3. Purified DNA (3 µg) was processed with methylEasy Xceed Rapid DNA Bisulfite Modification Kit (Human Genetic Signatures, Sydney, Australia). Robo4 promoter fragments were then amplified by PCR from bisulfite-treated DNA using position-specific primers (Supplementary Table S1), cloned into a vector using TOPO TA Cloning Kit (Invitrogen, Carlsbad, CA), and propagated in DH5α cells. Plasmids isolated from 10 randomly-picked colonies were sequenced.

### Preparation of Expression and Adenovirus Vectors

cDNA fragments of SOX7, SOX17, SOX18, and VEZF1 were amplified by PCR from HUVECs using specific primers (Supplementary Table S1). cDNA fragments of ETV2, TET1, and TET2, were also amplified by PCR from plasmids pENTR2223.1-ETV2 (DNAFORM, Kanagawa, Japan), pFN2.1-Halotag-TET1, and pFN2.1-Halotag-TET2 (Promega, Madison, WI) using specific primers (Supplementary Table S1). These fragments were cloned into pcDNA3 and pHMEF5^34^, an adenovirus shuttle vector in which inserts are controlled by the EF-1α promoter. pHMEF5-HA-ETV2 was generated by inserting a DNA fragment coding HA tag into pHMEF5-ETV2 using In-Fusion HD cloning kit (TAKARA Bio USA, Mountain View, CA). cDNA fragments of HA-TET1/2 were amplified by PCR from pHMEF5-TET1/2 using specific primers (Supplementary Table S1) and cloned into pHMEF5. The expression cassettes in the shuttle vectors were then inserted into the adenovirus parental vector pAdHM4, and amplified as described previously^35,36^. Briefly, HEK293 cells were transfected with linearized adenovirus vectors using Lipofectamine 2000 (Invitrogen), cultured, and lysed. Adenovirus vectors in cell extracts were then purified by centrifugation on a CsCl_2_ gradient, and vector titers were calculated by spectrophotometry^37^. Prepared adenovirus vectors infected COS7, HUVEC, and NHDF with more than 90% efficiency.

### Real-Time RT-PCR

Total RNA was prepared using RNeasy Mini Kit (Qiagen, Hilden, Germany) from undifferentiated and differentiated iPS cells, and from HUVECs infected with 3,000 adenovirus particles/cell, and from fibroblasts infected 10,000 adenovirus particles/cell. Total RNA (50 ng) was reverse-transcribed with Superscript III reverse transcriptase (Invitrogen), and analyzed by real-time PCR using specific primers (Supplementary Table S1) and QuantiTect SYBR Green PCR Kit (Qiagen). Copy numbers were calculated from standard curves constructed using known amounts of plasmids that contain target sequences. Expression was normalized against GAPDH, and data were collected from at least three independent experiments.

### Reporter Assays

Luciferase plasmids containing Robo4 promoters were constructed as previously described^26,27^. HEK293 cells (7.5 × 10^5^ cells/well) were transfected with 0.3 µg reporter plasmid and 0.3 µg expression vector using Lipofectamine 2000 (Invitrogen), cultured for 24 h, and analyzed for luciferase activity using a luminometer. Data were collected from three independent experiments, each of which was performed in duplicate.

### Electrophoretic Mobility Shift Assay

Recombinant ETV2 was prepared using TNT Quick (Promega) coupled transcription/translation system and 1 µg expression vector. ^32^P-labeled oligonucleotide probes spanning each ETS motif (Supplementary Table S1) were mixed for 30 min at 4 °C with 3 µl recombinant ETV2, and analyzed by electrophoresis at 120 V for 2 h on a 4% native polyacrylamide gel in 0.5 × TBE buffer.

### Chromatin Immunoprecipitation Assay

HUVECs were infected with 10,000 adenovirus particles (vp)/cell for 2 days to express ETV2 -FLAG or GFP. NHDF were infected with 10,000 vp/cell for 3 days to express HA-ETV2 or HA-TET1/2 with ETV2 or GFP. These cells were crosslinked in a solution containing 50 mmol/L HEPES-KOH pH 7.5, 100 mmol/L NaCl, 1 mmol/L EDTA, 0.5 mmol/L EGTA, and 1% formaldehyde, and sonicated for 20 cycles at 15 sec/cycle using Sonifer Model 250 (Branson, Danbury, CT), with output control 2 and duty cycle 40%. The resulting extract was incubated with Dynabeads precoated with 5–10 µg control IgG or antibodies against FLAG (Sigma) or HA (Santa Cruz Biotechnology, Dallas, TX). DNA-protein complexes were collected with a magnet, and de-crosslinked in a solution containing 50 mmol/L Tris-HCl pH 8.0, 10 mmol/L EDTA, and 1% SDS. The resulting DNA was quantified by real-timePCR with specific primers (Supplementary Table S1).

### Immunoprecipitation Assay

COS-7 cells (1.5 × 10^6^ cells/well) were infected with 5,000 adenovirus particles/cell or transfected with plasmids to express TET1-FLAG or TET2-FLAG with or without ETV2, cultured for 48 h, and lysed in 400 µl lysis buffer containing 50 mmol/L Tris-HCl pH 7.4, 150 mmol/L NaCl, 1% Triton X-100, 1 mmol/L EDTA, and 1× Protease inhibitor cocktail (Roche, Mannheim, Germany). Lysates were immunoprecipitated using FLAG Immunoprecipitation Kit (Sigma Aldrich). Precipitates were eluted with 100 ng/µl FLAG peptide, and analyzed by western blot using antibodies against FLAG (Sigma) and ETV2 (Abcam, Cambridge, UK).

### Statistical analysis

The statistical significance of differences between means was determined by Student’s *t* test, Dunnett’s test, or Tukey-Kramer test.

## Electronic supplementary material

Supplemental data
