# Supplementary material for: ETV2-TET1/TET2 Complexes Induce Endothelial Cell-Specific Robo4 Expression via Promoter Demethylation
Source: Sci Rep. 2018 Apr 4;8:5653. doi: 10.1038/s41598-018-23937-8 (PMC5884809; doi:10.1038/s41598-018-23937-8)
Supplement: Supplementary file 1 — Supplemental data [file 41598_2018_23937_MOESM1_ESM.pdf]

Supplementary Information

ETV2-TET1/TET2 Complexes Induce Endothelial Cell-Specific Robo4 Expression via Promoter Demethylation

Toru Tanaka, Kohei Izawa, Yusuke Maniwa, Maki Okamura, Atsumasa Okada, Tomoko Yamaguchi, Keisuke Shirakura, Naoki Maekawa, Hayato Matsui, Kenji Ishimoto, Nobumasa Hino, Osamu Nakagawa, William C. Aird, Hiroyuki Mizuguchi, Kenji Kawabata, Takefumi Doi, Yoshiaki Okada

Supplemental Information

Supplementary Figures

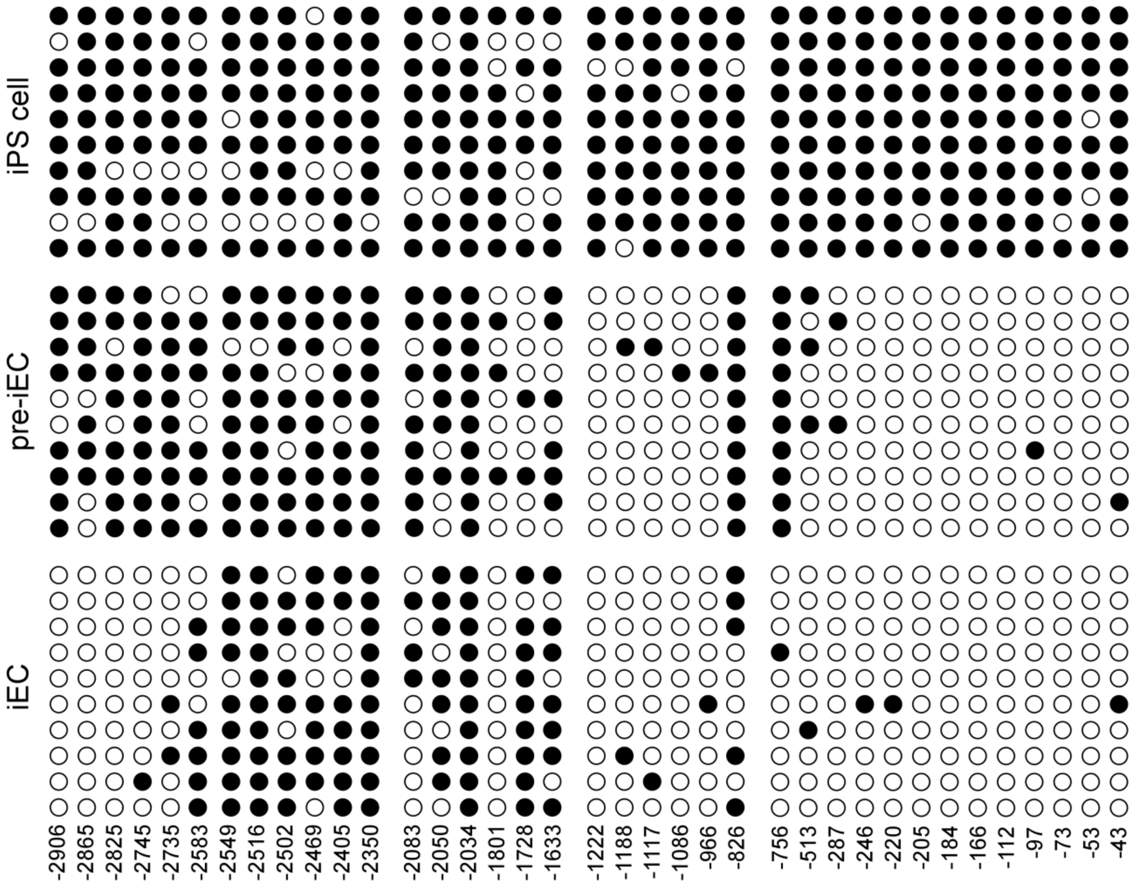

Figure S1. CpG methylation patterns of the Robo4 promoter in human iPS cells, pre-iECs, and iECs, as analyzed by bisulfite sequencing. Open and closed circles indicate non-methylated and methylated CpGs, respectively. Numbers indicate distance (base) from the transcription start site.

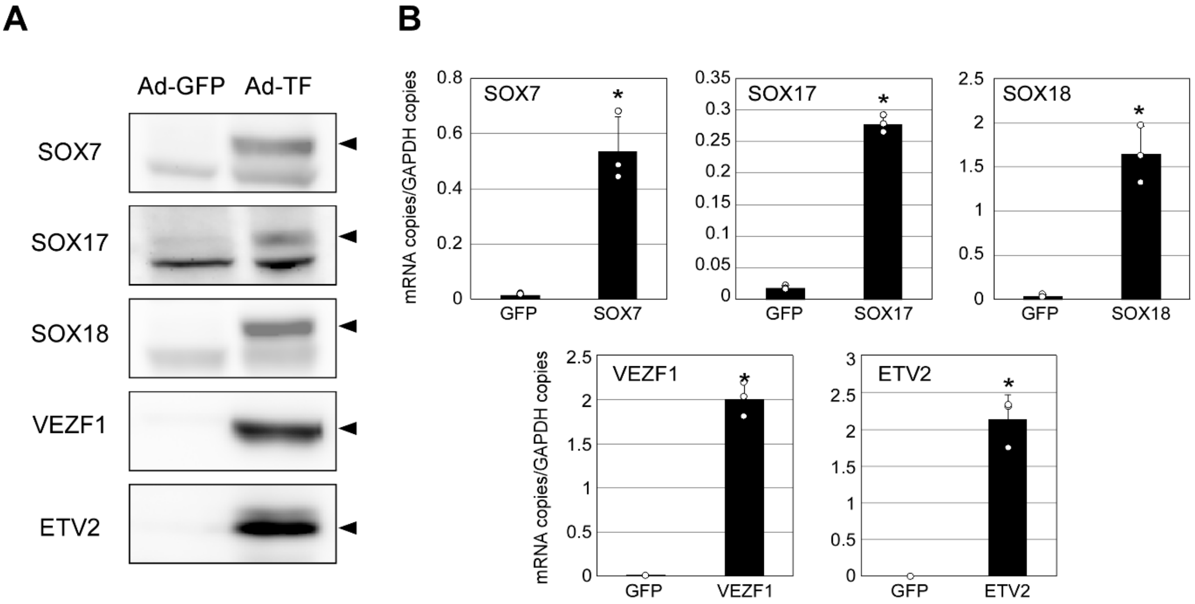

Figure S2. Expression of transcription factors from adenoviral vectors in endothelial cells. A, B, Protein (A) and mRNA (B) expression of transcription factors in adenovirus-infected HUVECs were analyzed by western blot and real-time RT-PCR, respectively. Data are means ± S.D. (n = 3). *, $p <$ 0.05 by two-tailed Student's t-test vs GFP.

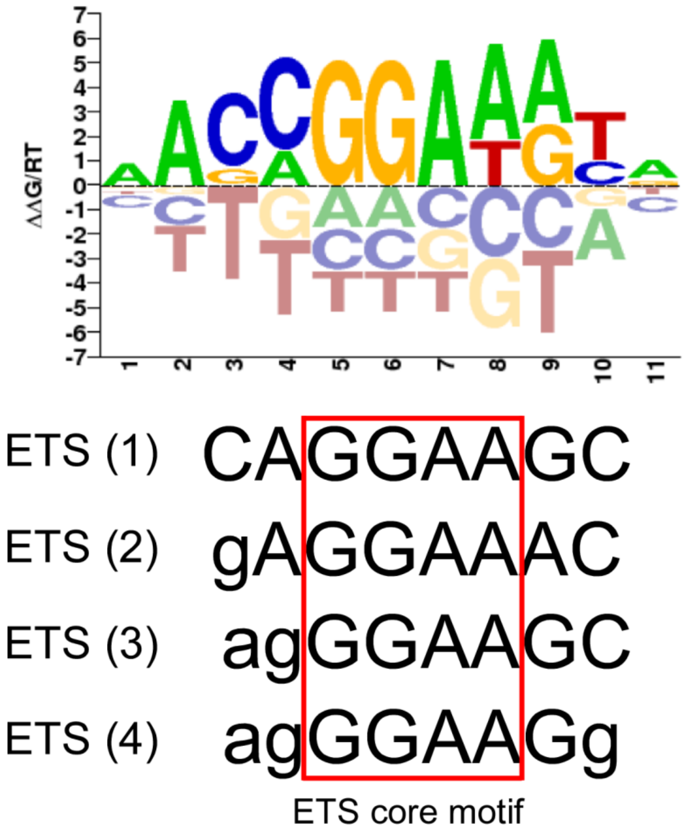

Figure S3. ETV2 consensus motif and ETS sites in the Robo4 promoter. The ETV2 consensus motif was obtained from the CIS-BP database and from a previous report[1]. The ETS(1)-(4) motifs in the Robo4 promoter are shown with the core motif (GGAA) and two flanking bases. Bases in lower case are not identical to the consensus motif.

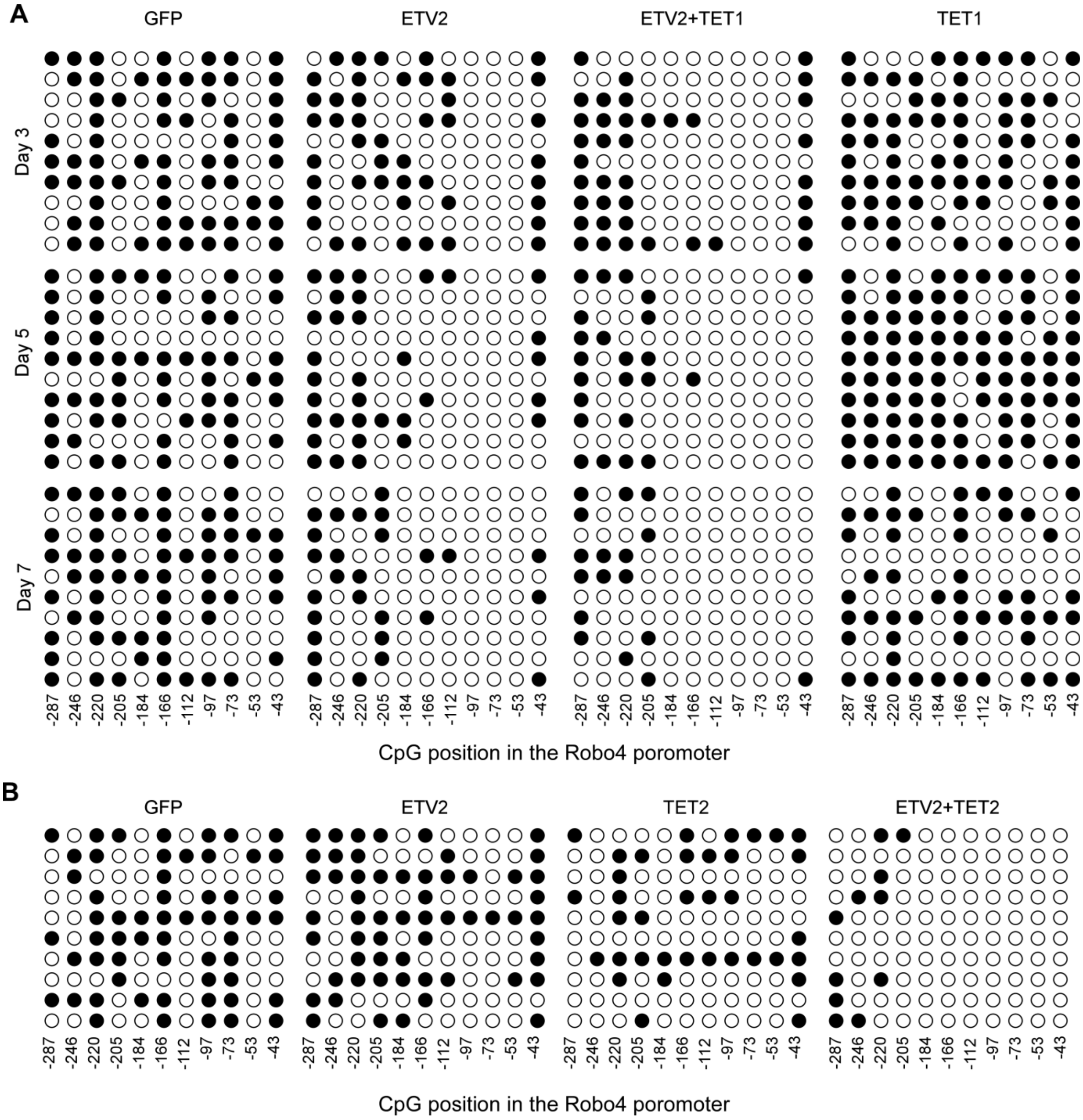

Figure S4. CpG methylation of the Robo4 promoter in human dermal fibroblasts expressing ETV2 or GFP with or without TET1/TET2. A, **B**, Open and closed circles indicate non-methylated and methylated CpGs, respectively.

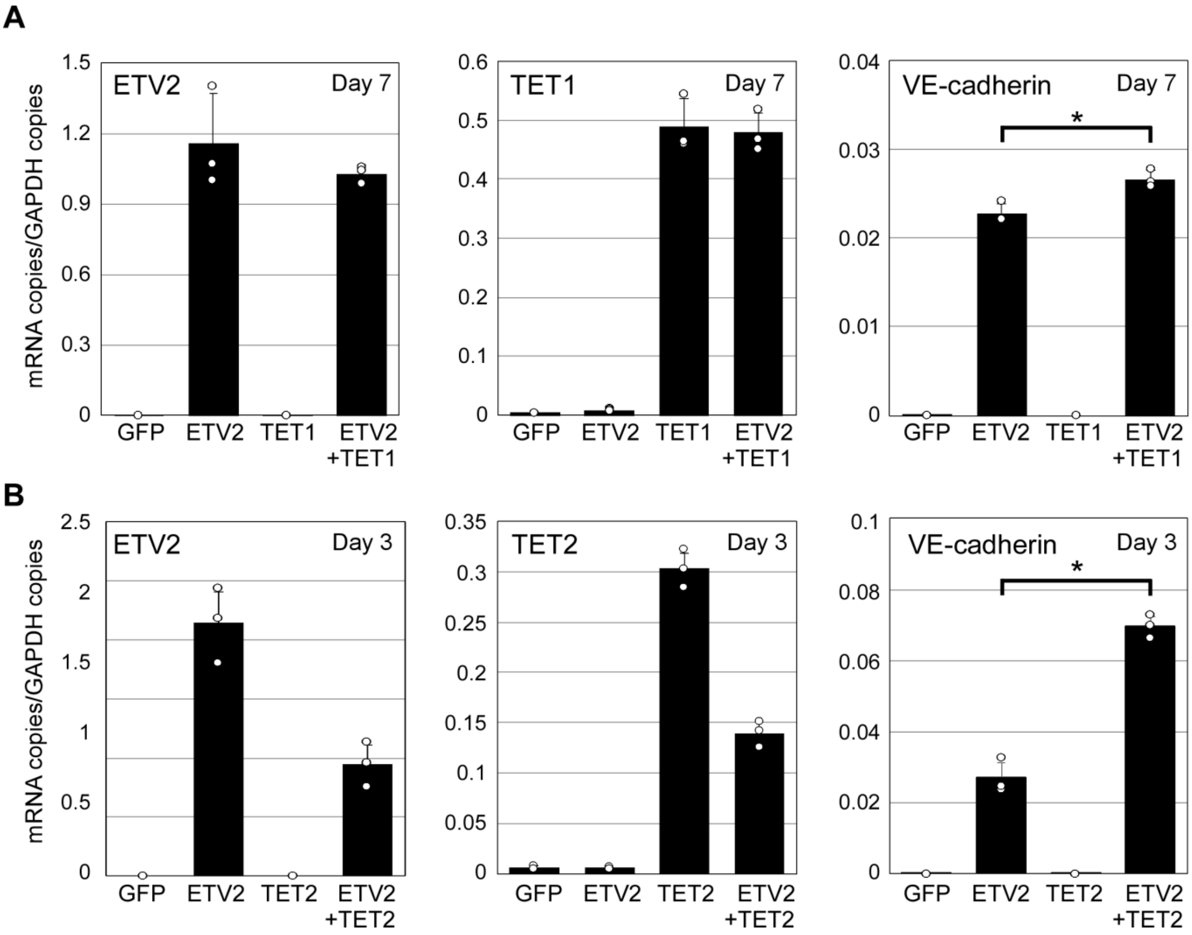

Figure S5. Expression of ETV2 and TET1/TET2 in fibroblasts expressing ETV2 with or without TET1/TET2. A, **B**, Fibroblasts were infected with adenovirus vectors on day 0 and day 3 to express either GFP or ETV2 with or without TET1 (A) and TET2 (B), and cultured for 3–7 days. Expression of ETV2, TET1, and TET2 was measured by real-time RT-PCR. Data are means ± S.D, (n = 3). *, $p < 0.05$ obtained from two-tailed Tukey-Kramer test.

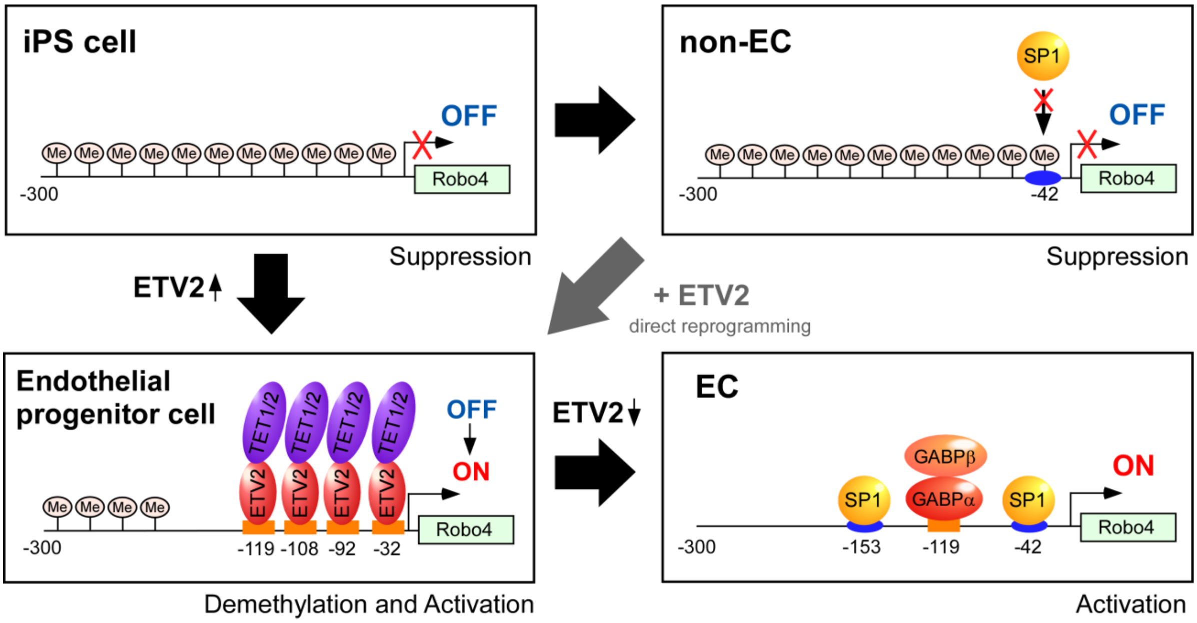

Figure S6. Endothelial cell-specific Robo4 expression via ETV2 and TET1/TET2. In undifferentiated iPS cells, the Robo4 promoter is highly methylated, and Robo4 expression is repressed. The methylation pattern is preserved by some mechanism during cell differentiation into non-ECs, and suppresses Robo4 expression by preventing transcription factors from binding. On the other hand, ETV2 is transiently expressed during differentiation into ECs, and induces promoter demethylation by recruiting TET1/TET2. Subsequently, ETV2 diminish with further differentiation into mature ECs, but GABP and SP1 bind to the non-methylated promoter to sustain Robo4 expression[2, 3].

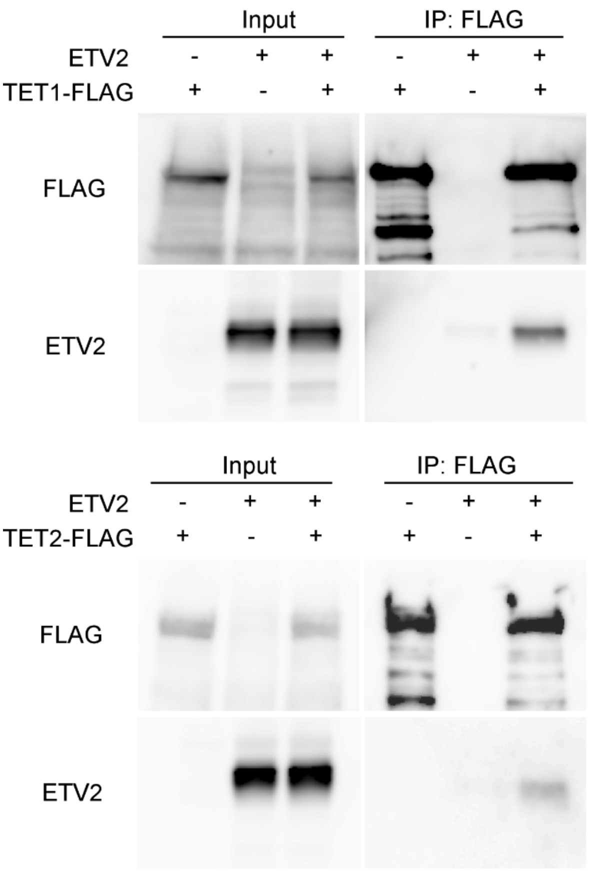

Figure S7. Full-length blots displayed in Fig. 5B and C.

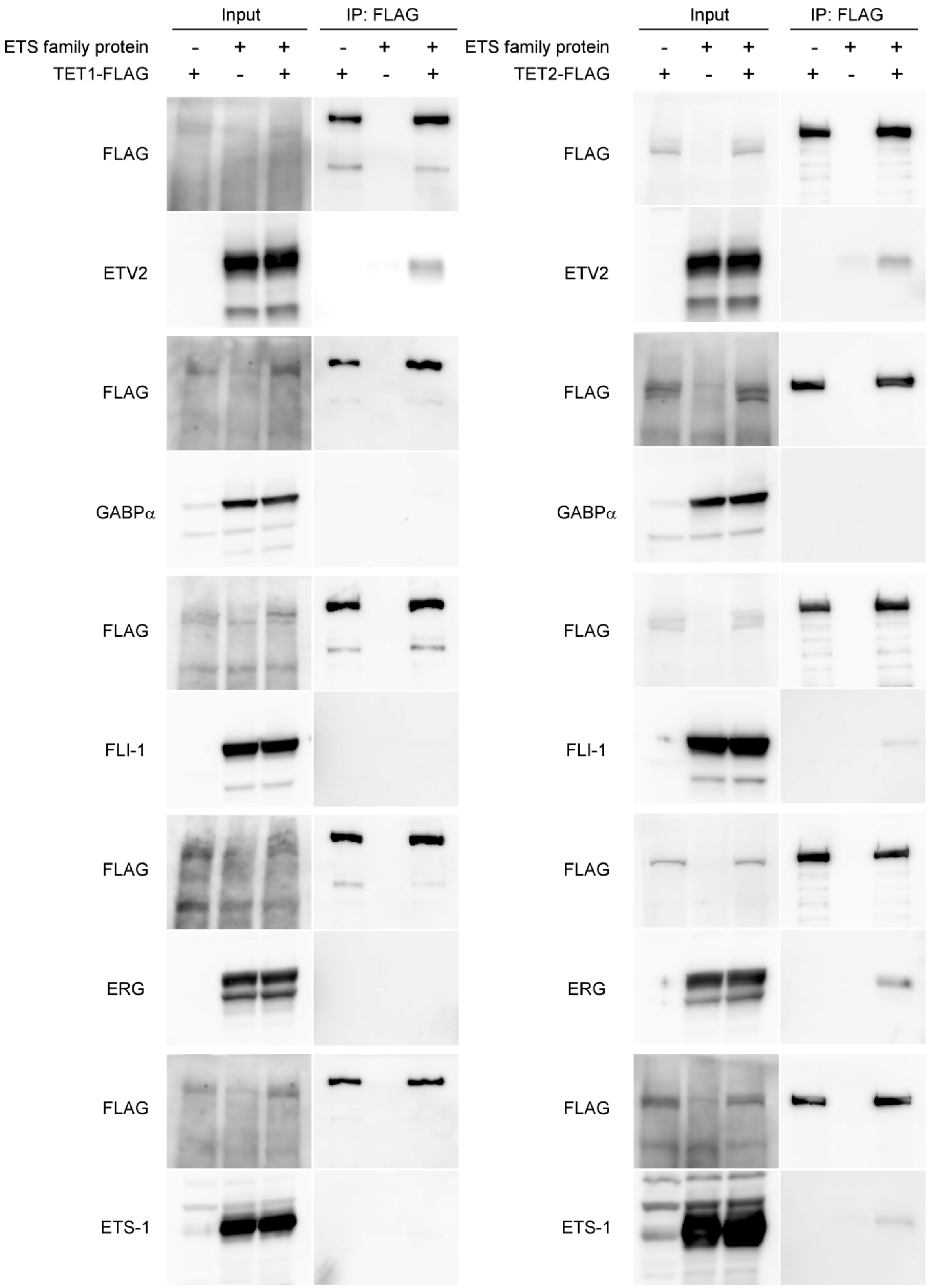

Figure S8. Full-length blots displayed in Fig. 7.

Supplementary References

1. Jolma, A. *et al.* DNA-binding specificities of human transcription factors. *Cell* **152**, 327-339 (2013).
2. Okada, Y. et al. A three-kilobase fragment of the human Robo4 promoter directs cell type-specific expression in endothelium. *Circ Res* **100,** 1712-1722 (2007).
3. Okada, Y. et al. A GABP-binding element in the Robo4 promoter is necessary for endothelial expression in vivo. *Blood* **112,** 2336-2339 (2008).

Supplementary Table

Supplementary Table S1. Primers and probes

**cDNA cloning and plasmid construction**		
SOX7	forward	5'-GGGGGTACCGTGCGAGGGCCAGGTCCG-3'
	reverse	5'-GGGGCGGCCGCGTGGGAGGAAAGCTGGTGTG-3'
SOX17	forward	5'-GGCGGTACCCTCAGTGCCTCACTCCCCAC-3'
	reverse	5'-GCGCTCGAGGTGTAACACTGCTTCTGGCC-3'
SOX18	forward	5'-GCCGGTACCCTCCCGCGCTCCGTTCCG-3'
	reverse	5'-GCGCTCGAGCTAGGGGGCTGTGACATGGA-3'
VEZF1	forward	5'-GCTTAGGATCCATGGAGGCCAACTGGACCGCGTTCC-3'
	reverse	5'-GCTTAGCGGCCGCTTACCAAGGCGGTGATGTAGGCAAA-3'
ETV2	forward	5'-GCCTGGATATCTATCAGAAGGCCTTCATCGCATCC-3'
	reverse	5'-GCCTGGCGGCCGCTTATTGTGTCTCTGCTCCCC-3'
TET1	forward	5'-CACACAGGTACCACCATGTCTCGATCCCGCCATGC-3'
	reverse	5'-CACACAGCGGCCGCAACGACCCAATGGTTATAGG-3'
TET2	forward	5'-GCTTGGTACCACCATGGAACAGGATAGAACCAACCATG-3'
	reverse	5'-GAGTGCGGCCGCTATATATCTGTTGTAAGGCCCTGTG-3'
HA-ETV2	forward	5'-TCTGCAGATCTATCCATGTACCCACACGATGTTCCAGATTACGCTGACC TGTGGAACTGG-3'
	reverse	5'-CCAGTTCCACAGGTCAGCGTAATCTGGAACATCGTATGGGTACATGGAT AGATCTGCAGA-3'
HA-TET1	forward	5'-CGGCGGTACCCACCATGTACCCATACGATGTTCCAGATTACGCTTCTCG ATCCCGCCATGCAA-3'
HA-TET2	forward	5'-TCCATGTACCCATACGATGTTCCAGATTACGCTGAACAGGATAGAACCA ACCATGTTG-3'
**Bisulfite sequencing**		
-802 to +16	forward	5'-GATGGGATGGGTTTAAAAAGAAATATAT-3'
	reverse	5'-ACCCATAACTACTCTCAACCCTATATC-3'
-1259 to -817	forward	5'-ATTTTTATTTGGTAGGTTGTTAGTTTGTGGTTG-3'
	reverse	5'-TTTTAAACCCATCCCATCACTAAAACTATTAAA-3'
-2297 to -1252	forward	5'-GTAGATTGTTGATAGTGATATTTTTGATAAGTTG-3'
	reverse	5'-AACCACAAACTAACAACCTACCAAA-3'
-3030 to -2347	forward	5'-AGGGGGAGTTAGAAAATATAAAATAT-3'
	reverse	5'-ACATTAATTTTAAAAACACTAATTAAATAC-3'
**EMSA**		
ETS(1)	sense	5'-GGGCCAGGCAGGAAGCATCGG-3'
	antisense	5'-CCGATGCTTCCTGCCTGGCCC-3'
ETS(1)mut	sense	5'-GGGCCAGGCAcctAGCATCGG-3'

	antisense	5'-CCGATGCTaggTGCCTGGCCC-3'
ETS(2)	sense	5'-CATCGGTTTCCTCAAAGCCGC-3'
	antisense	5'-GCGGCTTTGAGGAAACCGATG-3'
ETS(2)mut	sense	5'-CATCGGTTagGTtcAAGCCGC-3'
	antisense	5'-GCGGCTTTGAcctAACCGATG-3'
ETS(3)	sense	5'-CAAAGCCGCTTCCCTCCTGGG-3'
	antisense	5'-CCCAGGAGGGAAGCGGCTTTG-3'
ETS(3)mut	sense	5'-CAAAGCCGCTaggCTCCTGGG-3'
	antisense	5'-CCCAGGAGcctAGCGGCTTTG-3'
ETS(4)	sense	5'-CCCCTCCTTCCCTCTTCACTG-3'
	antisense	5'-CAGTGAAGAGGGAAGGAGGGG-3'
ETS(4)mut	sense	5'-CCCCTCCTaggCTCTTCACTG-3'
	antisense	5'-CAGTGAAGAGcctAGGAGGGG-3'
		**Real-time PCR**
CD31	forward	5'-CAGTGTCCCCAGAAGCAAAATAC-3'
	reverse	5'-CTCCGATGATAACCACTGCAATAA-3'
VE-cadherin	forward	5’-GCGACTACCAGGACGCTTTCA-3’
	reverse	5’-CATGTATCGGAGGTCGATGGTG-3’
Robo4	forward	5'-TTATGGCTCCCTCATCGCTG-3'
	reverse	5'-GAGGCTGTCTGAGCTGGAAC-3'
SOX7	forward	5'-GCCAAGGACGAGAGGAAAC-3'
	reverse	5'-TGACGCTGTCCCAGAAGAG-3'
SOX17	forward	5'-CAGAATCCAGACCTGCACAA-3'
	reverse	5'-CTTCGTGGAGGAGGCAGA-3'
SOX18	forward	5'-CTTCATGGTGTGGGCAAAG-3'
	reverse	5'-AAGCGTGGAAGGAGCTGAAC-3'
VEZF1	forward	5'-GACAGCAGCCGAACTTCGTT-3'
	reverse	5'-TGCCCGAGGAAGATGTAGTGA-3'
ETV2	forward	5'-CAGCTCTCACCGTTTGCTC-3'
	reverse	5'-AGGAACTGCCACAGCTGAAT-3'
TET1	forward	5'-AATGGAAGCACTGTGGTTTG-3'
	reverse	5'-ACATGGAGCTGCTCATCTTG-3'
TET2	forward	5'-GTGAGATCACTCACCCATCG-3'
	reverse	5'-CAGCATCATCAGCATCACAG-3'
TET3	forward	5'-GAGGAGCGGTATGGAGAGAA-3'

	reverse	5'-AGTAGCTTCTCCTCCAGCGT-3'
GAPDH	forward	5'-TGCACCACCAACTGCTTAGC-3'
	reverse	5'-GGCATGGACTGTGGTCATGAG-3'
**ChIP assay**		
-149 to -10	forward	5'-TCTGGTCTCCTCCCAGTTCTCCAAG-3'
	reverse	5'-CGAGCACTTTGTCCTGCTGCTCTG-3'
